# Supplementary figures and images for: Mortality in Switzerland in 2021
Source: PLoS One. 2022 Sep 9;17(9):e0274295. doi: 10.1371/journal.pone.0274295 (PMC9462753; doi:10.1371/journal.pone.0274295)

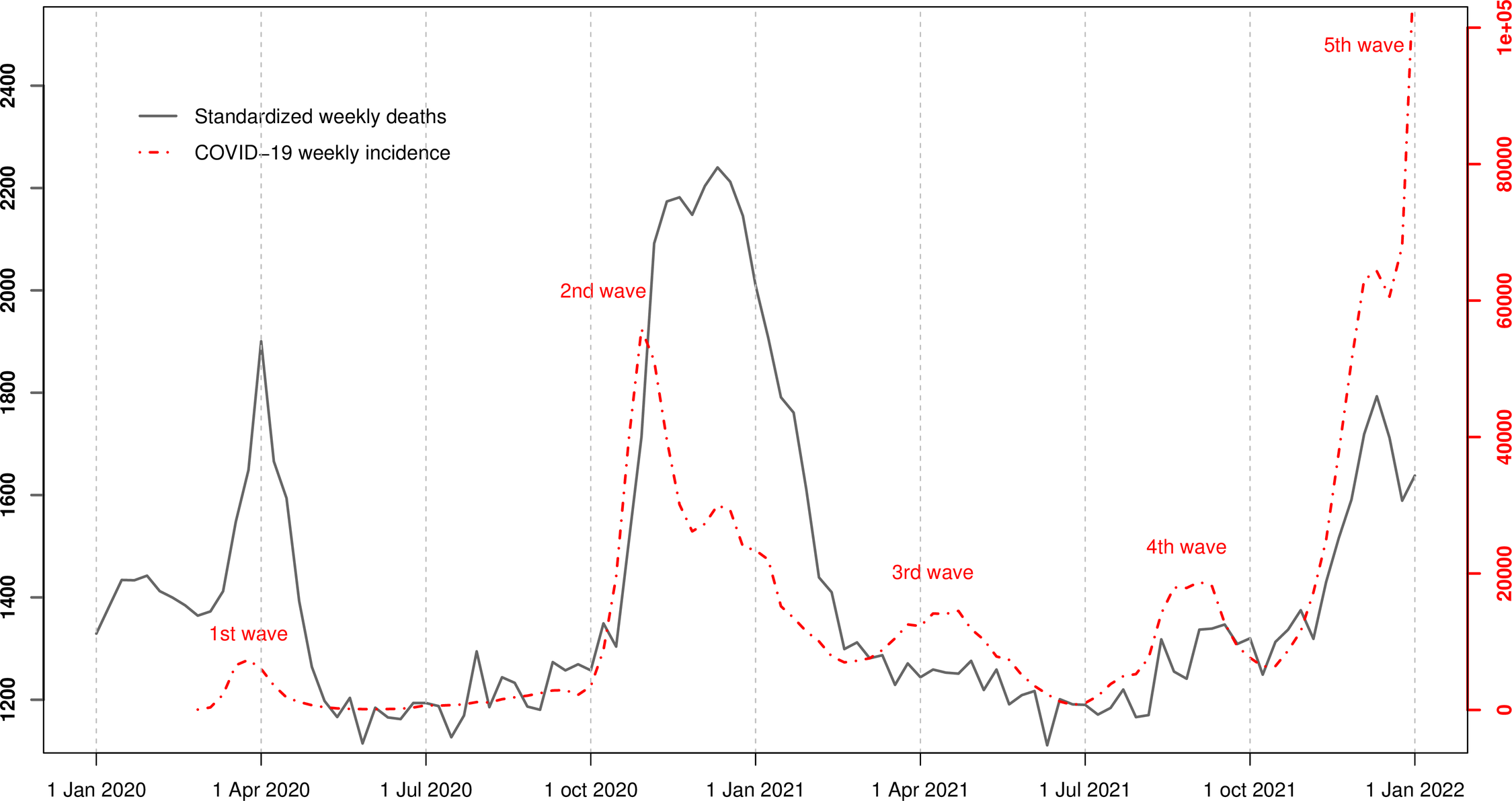

Supplement: S1 Fig — (TIF) [file pone.0274295.s001.tif]
