# Supplementary material for: Mortality in Switzerland in 2021
Source: PLoS One. 2022 Sep 9;17(9):e0274295. doi: 10.1371/journal.pone.0274295 (PMC9462753; doi:10.1371/journal.pone.0274295)
Supplement: S1 Appendix — (DOCX) [file pone.0274295.s002.docx]

# S1 APPENDIX

# Life expectancy in a piecewise exponential model

Let $l_{xj}^{y}$ be the probability of surviving to age $x$ ($x=0,\ldots,111$) for year $y$ ($y=2000,\ldots,2021$) and sex $j$ ($j=M,F)$. Such quantities were estimated according to a piecewise exponential model, i.e. with $l_{0j}^{y}=1$ and:

$$\begin{aligned} l_{xj}^{y}=exp\left( -\sum_{i=0}^{x-1} \dot{m}_{ij}^{y} \right) \# \left( A1 \right) \end{aligned}$$

where $\dot{m}_{ij}^{y}$ ($i=0,\ldots,110$) is the observed mortality rate in year $y$ for sex $j$ and for the 5-year age class (or the last open class of 90+) that age $i$ belongs to.

Life expectancy at age $x$ (sex $j$ and year $y$) was then obtained as the area under the $l_{tj}^{y}{/l}_{xj}^{y}$ $(t\geq x)$ conditional survival curve using a trapezoidal rule, as done in classical mortality table calculations [1], and taking 110 as the maximum age (technically, $l_{111j}^{y}=0$ and $\dot{m}_{110j}^{y}=\infty$) :

$$e_{xj}^{y} =\frac{1}{l_{xj}^{y}}\sum_{t=x}^{110} \frac{l_{tj}^{y}+l_{(t+1)j}^{y}}{2}$$

$$=\frac{1}{l_{xj}^{y}}\left\{ \frac{l_{xj}^{y}+l_{\left( x+1 \right)j}^{y}}{2}+\frac{l_{\left( x+1 \right)j}^{y}+l_{\left( x+2 \right)j}^{y}}{2}+\ldots+\frac{l_{109j}^{y}+l_{110j}^{y}}{2}+\frac{l_{110j}^{y}+l_{111j}^{y}}{2} \right\}$$

$$\begin{aligned} =\frac{1}{2}+\frac{l_{\left( x+1 \right)j}^{y}}{l_{xj}^{y}}+\frac{l_{\left( x+2 \right)j}^{y}}{l_{xj}^{y}}+\ldots+\frac{l_{110j}^{y}}{l_{xj}^{y}}. \#(A2) \end{aligned}$$

Plugging the piecewise exponential probabilities (A1) into (A2), one obtains the following expression for life expectancy at age $x$ (sex $j$ and year $y$):

$$e_{xj}^{y}=\frac{1}{2}+\exp\left( -\dot{m}_{xj}^{y} \right)+\exp\left( -\dot{m}_{xj}^{y}-\dot{m}_{(x+1)j}^{y} \right)+..+ \exp\left( -\dot{m}_{xj}^{y}-\dot{m}_{\left( x+1 \right)j}^{y}-\ldots-\dot{m}_{110j}^{y} \right)$$

$$=\frac{1}{2}+\sum_{l=x}^{110} \exp\left( -\sum_{i=x}^{l} \dot{m}_{ij}^{y} \right).$$

# Reference

1.   Shryock HS, Siegel JS (1976) The Methods and Materials of Demography. ACADEMIC PRESS, INC. Harcourt Brace Jovanovich, Publishers. San Diego, California.
